# Establishing a Theory-Based Multi-Level Approach for Primary Prevention of Mental Disorders in Young People

**DOI:** 10.3390/ijerph17249445

**Published:** 2020-12-16

**Authors:** Giuseppina Lo Moro, Emma Soneson, Peter B. Jones, Julieta Galante

**Affiliations:** 1 Department of Public Health Sciences, University of Turin, 10126 Torino, Italy; giuseppina.lomoro@unito.it; 2 Department of Psychiatry, University of Cambridge, Cambridge CB2 0SZ, UK; es703@medschl.cam.ac.uk; 3 National Institute for Health Research Applied Research Collaboration East of England, Cambridge CB2 8AH, UK

**Keywords:** children, adolescents, mental health, common mental distress, interventions, public health, mental health disorders, prevention

## Abstract

The increasing prevalence of mental health disorders and psychosocial distress among young people exceeds the capacity of mental health services. Social and systemic factors determine mental health as much as individual factors. To determine how best to address multi-level risk factors, we must first understand the distribution of risk. Previously, we have used psychometric methods applied to two epidemiologically-principled samples of people aged 14–24 to establish a robust, latent common mental distress (CMD) factor of depression and anxiety normally distributed across the population. This was linearly associated with suicidal thoughts and non-suicidal self-harm such that effective interventions to reduce CMD across the whole population could have a greater total benefit than those that focus on the minority with the most severe scores. In a randomised trial of mindfulness interventions in university students (the Mindful Student Study), we demonstrated a population-shift effect whereby the intervention group appeared resilient to a universal stressor. Given these findings, and in light of the COVID-19 pandemic, we argue that population-based interventions to reduce CMD are urgently required. To target all types of mental health determinants, these interventions must be multi-level. Careful design and evaluation, interdisciplinary work, and extensive local stakeholder involvement are crucial for these interventions to be effective.

## 1. Our Current Approach to Tackle Rising Youth Mental Health Problems is Unsustainable

Concerns about young people’s mental health are growing. The latest data from the Global Burden of Disease Study reported a prevalence of mental health disorders of 9.72% (95% CI 8.81–10.73%) among people under 20 years old, with a higher prevalence above 10 years old [1]. Mental health disorders are among the largest contributors to disability, morbidity and mortality in young people, and suicide is the second leading cause of death for 10–24-year-olds [2,3]. 

Studies throughout the world suggest that mental health disorders and psychosocial distress in this specific age group are rising [4,5,6,7,8,9,10,11]. In 2014, a systematic review on time trends of adolescents’ mental health reported an increasing burden of internalising problems [5]. More recent studies have confirmed this trend, particularly regarding depressive symptoms [6,7,8,9], emotional and behavioural problems [10], self-harm, and suicide-related outcomes [7,8]. Rates of mental health disorders in younger children are likely also increasing [11]. However, findings in this younger group are inconsistent [5], possibly because the major health surveys have generally started from the age of 15 and there is little consensus regarding indicators for mental health for younger children [2]. Changes in individual vulnerability, family life, extra-familial influences on risk and wider cultural change are among the possible explanations for these trends [12]. For example, changes in the timing of puberty and sleep patterns, increasing rates of parental depression, more stressful educational experiences, more marked inequalities, and increasing consumerist and individualistic values and attitudes may be linked to observed changes in young people’s mental health [12].

Experiencing mental health problems as a young person increases the chances of re-experiencing them in the future. Common mental health disorders in the teenage years are often precursors of disorders in young adulthood [13], and around half of adult mental health disorders start in adolescence [14]. Given that persistence of adolescent disorders is a strong predictor for adult mental health disorders, clinical and preventative interventions to target early episodes may prevent subsequent mental health disorders [13]. 

Nevertheless, youth mental health services worldwide have too little capacity and too few resources to stem the tide of increasing mental illness. Disparities between need and access to mental health services are even greater in low- and middle-income countries [15]. The gaps in treatment for mood disorders, anxiety disorders, and obsessive-compulsive disorders are all higher than 50% [16]. The World Health Organization (WHO) Atlas on global child and adolescent mental health resources reported widespread shortages of mental health professionals, lack of training standards, and suboptimal resource allocation [16,17]. Taking the UK as an example, a 2016 report found that, on average, 28% of those referred to Child and Adolescent Mental Health Services (CAMHS) were declined by the service (up to 75% in some regions) and some areas had waiting times of up to 200 days [18]. Four in every five CAMHS accepted only those young people with the most severe presentations, leaving many others to “slip through the cracks”. In some cases, even the most severe presentations were not sufficient to merit immediate access: 14% of young people with a life-threatening condition (e.g., suicide, self-harm, or psychosis) were not accepted to any service and half were put on waiting lists. Throughout Europe, only three in five countries have early intervention youth mental health community services, 58% evaluate the quality of the services, and 52% have guidance to help facilitate the transition from child to adult services [19]. The lack of appropriate global resources for mental healthcare for young people demands new plans to fill the gap between research and practice regarding prevention: when the bathroom is flooding, we need the tools to turn off the water, not focus on mopping the floor.

## 2. Including Context as Well as Individual Risk and Resilience Factors

After decades of research, we know that the determinants of mental ill health are not just biological [20]. Non-biological determinants of mental health disorders include demographic, social, cultural, economic, and environmental factors (Table 1). Prenatal environment, birth complications and preterm delivery already have effects on mental health [21]. Demographic factors such as gender and age also have a role [22,23,24]. Childhood and adulthood adversities, stressful life events, and trauma can substantially increase vulnerability to mental health disorders [21,22,24,25,26]. Poor family relationships and connectedness, peer social connections, social support, and community belonging are other key determinants [22,23,25,26,27]. In addition, discrimination, whether linked to ethnicity, immigrant status, sexual orientation, gender identity, or occupational status, is associated with negative mental health outcomes [27]. Economic factors, such as socio-economic disadvantage, poverty, financial strain, and income inequality are widely reported to influence mental health [21,23,24,25,26,27,28,29], along with employment conditions [22,27], food insecurity [26,30], and housing instability [26]. Last, urbanicity, neighbourhood safety, air pollution, and climate change each impact mental health [21,23,27,31]. This body of evidence clearly supports a multi-level causation model that is not unique to mental health, but rather reflects well-established ideas around the social determinants of physical health. 

Several existing bio-psycho-social models help add structure to these many influences. For example, the existence of multiple levels of influence on human development is at the centre of Bronfenbrenner’s bioecological model, in which Process, Person, Context, and Time shape human development. The core of the model consists of proximal processes, which are forms of interaction between the person and the surrounding environment; these processes are the “engines” of development. The power of these processes on development can vary as “a function of the characteristics of the developing Person, of the immediate and more remote environmental Contexts, and the Time periods, in which the proximal processes take place” [32] (p. 795). The importance of multiple layers of development is also considered in other theories, such as Krieger’s ecosocial approach or Dahlgren and Whitehead’s “rainbow” model of the determinants of (ill) health, each of which highlights the importance of a variety of influences ranging from an individual’s genes to overarching social structures and cultural norms [33,34].

These bio-psycho-social models are not limited to risk factors for poor mental health, however; they can also help us to conceptualise resilience to stress as a result of the interaction between the individual and multiple reciprocating systems. According to Ungar’s model of resilience, proximal processes can drive different but equally viable developmental trajectories (equifinality). The influence of these proximal processes can vary according to characteristics such as nature of risk or available resources (differential impact); different contexts and cultures lead to different processes (contextual and cultural moderation) [35]. In addition, Tol and colleagues (2013) examined children and adolescents facing specific adversities (e.g., armed conflict) and showed that factors that promote and protect mental health may result from multi-level interactions involving individual, family, peer, school, and community levels. For instance, coping style and personal strengths, parental support and monitoring, school retention, community acceptance, and social support might interact to determine mental health and wellbeing [36].

Furthermore, early onset of mental health disorders generates damage that exacerbates unfavourable conditions and perpetuates them across generations, leading to “vicious cycles” of intergenerational risk. This damage is exerted not only in the form of morbidity and early mortality for the individual, but also at the societal level in terms of missed opportunities and increased costs for health services and related sectors. Accordingly, the benefits of better mental health in young people extend far beyond health to areas such as education, employment and criminal justice [37]. 

Social and environmental determinants of health are thus key contributors to the global burden of mental health disorders. It becomes evident that our approach to dealing with youth mental health disorders will only become sustainable when we encompass a contextual, bio-psycho-social approach to address these wider determinants.

## 3. The Covid-19 Pandemic Provides Further Evidence of Multi-Dimensional and Multi-Level Determinants of Mental Health

Sadly, we can take the COVID-19 pandemic as a ghastly natural experiment whereby a universal stressor is applied. Young people are among those displaying the largest increases in psychological distress and mental ill health [4,38]. Evidence shows that conditions related to COVID-19 and previous pandemics (e.g., quarantine and social isolation) are linked with acute stress disorder, post-traumatic stress disorder, behavioural problems, anxiety, and depression among young people [39,40,41,42,43]. 

An ecological view of the pandemic’s effects on mental health highlights stressors at many different levels (Table 1). There is some evidence that the virus could directly affect mental health. It is known that infections can be associated with greater risk of mood disorders and other mental health disorders; as such, the coronavirus may affect the brain directly (e.g., brain infection) or indirectly (e.g., massive cytokine response, hypoxic injury, or through a pro-coagulant state) [44,45,46]. However, the psychiatric sequelae of COVID-19 are likely due to the combination of several multi-level factors, including social isolation, fear of a new severe disease, socio-economic uncertainty, loss of loved ones, stigma, and fear of infecting others [46]. In fact, it would be unreasonable to ascribe the increase in common mental health disorders to a direct biological cause, since most of the community respondents will not have been infected. 

For young people in particular, the pandemic sets the stage for the onset or intensification of several risk factors related to poor mental health. Domestic violence, child maltreatment, social deprivation, accommodation issues, school closures, parental job loss, and exposure to frightful news are all on the rise [47,48,49,50]. The pandemic may have an even greater impact on mental health of young people who are already experiencing problems, for example, those with existing mental health disorders [47]. A survey of young people with histories of mental health disorders found that 83% declared that the pandemic had made their mental health worse and 26% declared that they were unable to access mental health support [51,52]. In particular, school closures decrease access to care for young people with mental health needs [51], as schools are often the most common mental health service setting for children and adolescents [53]. 

The extensive evidence on the deleterious effects that disempowerment, deprivation, inequity, job instability, low socioeconomic status, transition into income poverty during early childhood, uncertainty, lack of control, loneliness, and isolation can have on mental health seems more than sufficient to explain the increases in mental ill health arising during the pandemic [43,54,55,56,57,58,59,60,61,62]. In addition, inequalities in adverse experiences during the pandemic have been maintained with no decrease in discrepancies between socioeconomic subgroups [63]. All considered, the COVID-19 pandemic is a stark reminder that most of the triggers for mental health disorders are psychosocial [64].

## 4. A Public Health Approach to Prevention: Shifting the Population Mean

In order to determine how best to address the multi-level risk factors for mental ill health, we must first understand the distribution of risk. More often than not, risk factors are distributed throughout the population, often “normally,” with most people hovering around the mean and only a few inhabiting either extreme. This raises a question as to which strategy is optimal for reducing risk: targeting the small group of individuals at highest risk (the “high-risk” approach), or aiming to “shift” the entire mean through smaller risk reductions across the whole population (the “population approach”).

In 1985, the epidemiologist Geoffrey Rose examined the advantages and disadvantages of the high-risk and population approaches to prevention [65]. Using findings from various physical health conditions, he proposed that the larger group of people at moderate or small risk may contribute more to the overall incidence of disease than the smaller group of people at high risk—an idea that would lend weight to the population approach to prevention. Indeed, Rose ultimately argued for the superiority of the population approach, viewing it as more “radical” (p. 37) than its “palliative and temporary” (p. 37) counterpart, though he acknowledged that the two approaches were not incompatible [65]. 

For other non-communicable disorders such as heart disease and stroke, population interventions that aim to prevent disease by shifting the group mean of key risk factors (e.g., blood pressure) are common. Population approaches have been less developed for mental health disorders, and there is controversy around whether the so-called Rose hypothesis applies to prevention of mental health disorders [66]. However, there has been considerable progress over the last decades, with reductions in the onset of some disorders having been found as a result of primary prevention programmes [67]. These successes may be explained by the Rose hypothesis, as illustrated below.

Let us take common mental distress (CMD) as a risk indicator for mental health disorders. CMD, sometimes called the psychopathology or “p” factor, is a psychometrically-derived common factor across scales measuring a wide range of psychiatric symptoms such as depression, anxiety, psychosis, etc. [68]. As such, CMD measures levels of mental distress rather than focusing on any particular set of symptoms and therefore transcends the diagnostic categories traditionally used in psychiatry. It has a normal distribution in the general population and attends to cross-cultural differences as much as the scales informing CMD do. The higher an individual scores on this factor, the worse the individual “fares on indicators tapping severity, duration of disorder, extent of sequential comorbidity, adult life impairment, childhood developmental history, family history of liability to psychiatric illness, and brain function from early life to midlife” (p. 14) [68]. 

**Figure 1:**
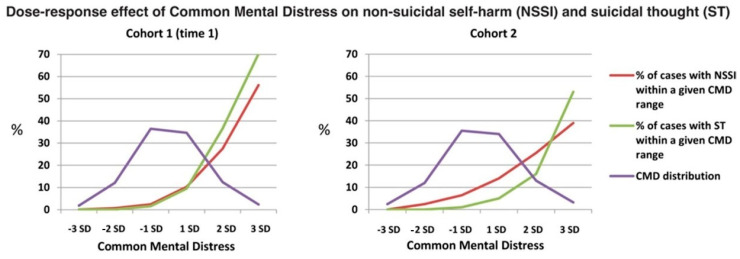
Dose-response effect of common mental distress on non- suicidal self- injury (NSSI) and suicidal thought (ST) in cohort 1 and cohort 2. The normal population distribution of CMD, which was strikingly similar, but not identical, in cohorts 1 and 2, is shown by the purple line. CMD, common mental distress. (Reproduced from Polek 2020 [69] with permission).

We have collaborated with Polek and colleagues [69], who used data from two independent UK population-based cohorts of volunteers aged 14–24 years (*N* = 2403 and 1074) to assess the risk of non-suicidal self-harm and suicidal thoughts across the distribution of CMD [69]. For this, they first used psychometric methods and a bifactor model to establish a robust, latent factor of depression and anxiety normally distributed across the population. Then, they plotted distributions of CMD scores in both cohorts against lines representing percentages of individuals reporting non-suicidal self-harm and suicidal thoughts within bands of a normally distributed CMD expressed as standard deviations. Figure 1 shows the dose-response relationship that they found, replicated in both cohorts, between CMD and risk of these two severe symptoms. More than two-thirds of the individuals experiencing these symptoms had CMD scores between two standard deviations above the population mean, and around half had scores within one standard deviation. Very high CMD scores indicated the highest risk but were rare, and so generated relatively few events.

As well as CMD, all of the domains of mental health that Polek et al. measured (namely depression, anxiety, self-esteem, well-being, psychotic-like experiences, antisocial and schizotypal traits, conduct problems, obsessions, and compulsions) predicted risk of self-harm and suicidal thoughts. The beauty of CMD was, however, its efficiency as a normally-distributed summary measure of the damaging effect that these domains have in common.

In support of using CMD, accumulating empirical studies suggest that many symptoms conventionally seen as components of distinct mental health disorders manifest as a single, latent dimension that is distributed within the general population [70,71]. For example, low-prevalence events such as psychotic experiences are better predicted by models using this dimensional approach than by those using distinct mental health disorders [68]. Support also comes from longitudinal data analyses. For example, Polek et al. found that CMD mediated the persistence of self-harm and suicidal thoughts over two years [69]. The latent CMD model fits better with several observations in mental health: high comorbidity of psychiatric diagnoses, shared causal factors and treatments, and transdiagnostic psychological and neural correlates [72]. 

Taken as a whole, this evidence supports the idea that psychopathological items accumulate in a probabilistic manner rather than in diagnostic clusters, with common symptoms of depression and anxiety generally occurring before rarer phenomena such as suicide or psychotic experiences. Less frequent manifestations may show up as clusters as CMD increases, with these clusters giving rise to disorder-specific medicine and epidemiology.

Thus, we should prioritise universal preventative interventions aimed at lowering the mean CMD in a population (i.e., “shifting the curve”) rather than targeting those with mental health disorders. Prevention policies that embrace the whole population have other advantages: they do not depend on disorder screening and identification, so they do not encounter problems derived from stigma, data collection problems or inadequate risk assessment. Therapy and targeted prevention are still important, but should not be our only approaches.

We have obtained very encouraging results from a universal strategy to reduce psychological distress among a sub-population of young people that is becoming increasingly numerous: university students [73]. The Mindful Student Study was a pragmatic randomised controlled trial assessing the provision of an eight-week course on mindfulness skills for students (MSS) that aimed to increase University of Cambridge students’ resilience to stress. The main outcome was self-reported CMD while revising for their yearly exams—another universal stressor—as measured by the CORE-OM questionnaire [74]. Over 600 students took part and were randomised to being offered the MSS course plus mental health support as usual, or to support as usual alone [75].

**Figure 2:**
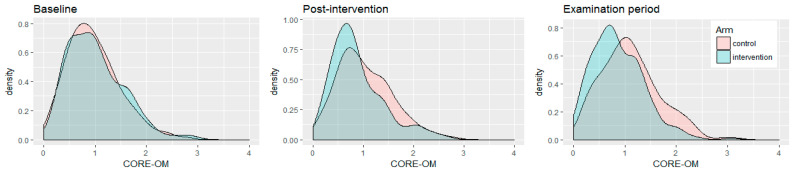
Density plot illustrating the CORE-OM psychological distress scores for each trial arm as students progressed from the moment they signed up for the study (baseline), 2 months later (post-intervention), and during the examination period (4–6 months later). CORE-OM total mean scores range from 0 (no distress) to 4 (maximum distress) (reproduced from Galante 2018 [73] with permission).

The Mindful Student Study has shown that, on average, the MSS reduced students’ examination period CMD by a moderate but clinically significant amount compared with the control group. Figure 2 illustrates that while the CMD distributions of each trial arm were indistinguishable at baseline (consistent with participants having been randomly assigned to arms), CMD subsequently increased in the control arm but not in the MSS arm, to the point that these distributions became different beyond chance effects (*p* < 0.001). By maintaining average CMD levels throughout a stressful period, the MSS effectively shifted the CMD population mean to the left [73]. Other interventions promoting mental health among young people may achieve this, too (for example, the intervention by Shinde et al. [76]), and educational institutions are particularly attractive as their settings [77].

## 5. Public Mental Health Prevention to Tackle Individual and Social Determinants

Another commendable aspect of population-based preventative interventions for non-communicable physical diseases is that they aim to shift the group mean by targeting determinants at individual, social, and systemic levels. For example, for preventing cardiovascular disease, and just looking at one determinant (smoking), interventions range from psychological support to quit smoking to tobacco taxes, regulated advertising, and smoking bans in public spaces. Only when they were combined was a large impact realised [78,79]. The MSS is an example of a successful intervention at the individual level, but it is unlikely to have a large impact if it is not combined with a) other interventions at that level to target other determinants and b) interventions at other levels to target distress and other determinants.

Multi-level interventions are designed to execute actions that target upstream determinants of health at more than one level of organisation, for example, by both delivering parenting workshops *and* implementing policies to facilitate engagement such as paid leave or childcare. Multi-level interventions constitute a very promising avenue for primary prevention of mental health disorders among young people, but well-designed and carefully-evaluated multi-level interventions are still scarce in mental health promotion and prevention [80,81]. 

Typical of a developing field, methods for effectiveness evaluation also need to improve [82]. Multi-level interventions are more resource-intensive but not always superior to single-level ones [83]. The multiple actions inherent in multi-level interventions should have multiplicative or summative effects; for this, actions should be modelled as mediators and moderators of effect based on mechanistic understandings [84]. In the simple multi-level intervention example given in the above paragraph, policies moderate the effect of workshops because the success of the latter will depend on the former. Multiple pathways with serial or parallel actions may be needed. 

Special care should be taken to tailor interventions to specific contexts [85,86] rather than using one-size-fits-all solutions imposed with a top-down approach. Actions must target the most locally relevant determinants of mental ill health, be they biological, psychological, or social, so interdisciplinary work is needed [87]. Genuine partnerships with local community representatives, policy-makers, and young people themselves [88,89], as well as creative and interdisciplinary use of existing resources (e.g., schools [77,90]), can help ensure interventions are locally feasible, acceptable, engaging, and sustainable.

The complexity of the efforts that are needed should not make us shy away from the problem [91]. Problem oversimplification facilitates intervention in the short term but is detrimental in the long term [92]. Research in this space needs to be highly interdisciplinary and collaborative; structured approaches that guide knowledge sharing among researchers across domains are needed [93]. 

## 6. Conclusions

In summary, we call for empirically-informed and theory-based interdisciplinary programmes of research and implementation of multi-level interventions that focus on universal prevention of youth mental health disorders by tackling determinants at individual, social, and systemic levels. These programmes must work with and receive full backing from all sectors of society, crucially from governments. We know that this is not an easy feat, reflected in the fact that global organisations such as the World Health Organization have made similar calls before [62]. However, the global urgency could not be greater than it is currently, and we collectively have the tools and knowledge to progress the field.

## Figures and Tables

**Table 1:** Main non-biological determinants of mental health disorders.

Determinant	Demographic	Socio-Cultural	Economic	Environmental
COVID-19 unrelated	Gender	Adversity and stressful life events	Socio-economic disadvantage	Urbanicity
	Age	Poor family, peer, and community connections	Poverty	Neighbourhood safety
		Discrimination	Financial strain	Air pollution
			Income inequality	Climate change
			Employment conditions	
			Food insecurity	
			Housing instability	
COVID-19 related		Social isolation	Job loss or instability	
		Social deprivation	Socio-economic uncertainty	
		Loss of loved ones	Inequity	
		Domestic violence and child maltreatment during isolation periods	Transition into income poverty during early childhood	
		Stigma	Accommodation issues	
		Fear of infecting others		
		Exposure to frightful news		
		Lack of control		
